# The role of ozonized oil and a combination of tobramycin/dexamethasone eye drops in the treatment of viral conjunctivitis: a randomized clinical trial

**DOI:** 10.1007/s10792-020-01503-4

**Published:** 2020-07-22

**Authors:** C. Cagini, M. Mariniello, M. Messina, A. Muzi, C. Balducci, A. Moretti, L. Levorato, A. Mencacci

**Affiliations:** 1 Division of Ophthalmology, Department of Surgical and Biomedical Sciences, University of Perugia, Ospedale S. Maria della Misericordia, S. Andrea Delle Fratte, 06156 Perugia, Italy; 2 Microbiology Unit, Department of Medicine, University of Perugia, Ospedale S. Maria della Misericordia, Perugia, Italy

**Keywords:** Viral conjunctivitis, Ozonized oil eye drops, Therapy, Inflammation

## Abstract

**Purpose:**

To determine whether topical tobramycin 0.3%/dexamethasone 0.1% plus ozonized oil eye drops reduces clinical signs and infectious viral titers of presumed viral conjunctivitis more than tobramycin/dexamethasone eye drops alone.

**Methods:**

Prospective, single-blind, randomized, parallel-groups trial. Eighty patients with a clinical diagnosis of presumed viral conjunctivitis were randomizedly divided into two treatment groups: a study group and a control group, 40 for each group. Patients in the study group received topical tobramycin 0.3%/dexamethasone 0.1% eye drops, plus ozonized oil eye drops, both four times daily; patients in the control group received only topical tobramycin 0.3%/dexamethasone eye drops four times daily. The treatment was for seven days in both groups. Swabs were taken from the conjunctival fornix for adenovirus PCR analysis on the day of recruitment and at seven days follow-up. Clinical signs were also recorded on the day of recruitment and at follow-up examination: the main outcomes were conjunctival injection and conjunctival chemosis, graded on a 4-point clinical scale, presence or absence of superficial punctate keratitis and subepithelial corneal infiltrates.

**Results:**

No statistically significant difference was reached in adenoviral infection negativization between the two groups, although the study group showed a higher number of PCR negative results at seven days follow-up. PCR real time detected adenoviral infection in 17 of 24 patients on the day of recruitment and it was positive in 4 patients on the seventh day (viral positivity reduction of 76%). In the control group PCR was positive for adenovirus in 18 of 24 patients on the day of recruitment and in 7 patients at seven days follow-up (reduction of 61%). There was statistically significant difference on conjunctival clinical signs between the study and control groups. Significant difference was also found on superficial punctate keratitis resolution between the study and the control group. In the former superficial punctate keratitis was detected in 14 eyes on the first day and in 5 eyes after seven days while in the latter superficial punctate keratitis was found in 124 eyes on the first day and in 6 eyes on the seventh day. No difference was found in subepithelial corneal infiltrates appearance between the two groups.

**Conclusions:**

The use of ozonized-oil containing eye drops in combination with topical tobramycin 0.3%/dexamethasone 0.1% eye drops four times daily seems to reduce the signs of conjunctivitis, and the duration of viral infection, although it does not affect the subepithelial corneal infiltrates appearance.

## Introduction

Acute viral conjunctivitis is a common, highly symptomatic and highly contagious infections mainly due to adenoviruses, with seasonal outbreaks, which causes significant discomfort and often lost working activity [1]. Although mostly self-limiting, in some cases it can lead to long term complications from immune-mediated sequelae [2]. There are no approved specific therapies for viral conjunctivitis although several therapeutic agents have been studied in both animal models and human trials [3–6]. In the absence of approved treatments, ophthalmologists recommend different therapeutic strategies that include artificial tears, cold compresses, topical antibiotics and topical steroids [7]. It is believed that the use of topical antibiotics can avoid bacterial superinfections, while short topical steroid cycles can limit patient discomfort and prevent some immune-related inflammatory complications. However, this therapeutic strategy is not approved by all because clinical studies indicate that a short course of topical corticosteroids without the addition of a suitable anti-viral agent can increase the duration of viral shedding and prolong the infectivity of the patients [8].

Furthermore, different ophthalmic formulations of dexamethasone in combination with povidone-iodine have been investigated for the treatment of viral conjunctivitis: both components are approved for use in other ophthalmic indications and have been shown to be safe using them on the humans ocular surface [9–12].

The recent introduction of ozonide eye drops for disinfection of the ocular surface is gaining attention among ophthalmologists. Oxidizing agents have long been used for their ability to disinfect the skin and their use offers essentially two advantages: they act against all microorganisms and do not induce resistance. Ozone gas (O3) is a molecule consisting of three oxygen atoms in a dynamically unstable structure [13] and is used to improve the repair of trophic ulcers, ischemic ulcers and diabetic wounds [14]. Moreover, thanks to its oxidative power, ozone is one of the best bactericidal, antiviral and antifungal agent and is used topically in the form of ozonated oil in numerous situations including for the treatment of wounds, anaerobic infections, herpetic infections [13]. Ozone, due to its anti-inflammatory and bactericidal activity as well as tissue repair, would find a very interesting field of application in infectious pathologies of the ocular surface.

A specific formulation has been recently developed for ophthalmic use, based on liposomal sunflower ozonated oil (Ozodrop®, FB Vision, Ascoli Piceno, Italy), and some authors have reported the tolerability of the conjunctival mucosa and ocular surface of ozone-based preparations [15], its efficacy in vitro and their benefit both in animals and humans [13].

To our knowledge there are no prospective studies in which ozone-based preparations have been tested in the treatment of infectious diseases affecting the anterior segment in humans. The aim of this pilot study was to verify if the use of ozonized oil eye drops added to the usual treatment in patients with clinical diagnosis of viral conjunctivitis may have an additional beneficial effect compared to the conventional treatment.

## Materials and methods

All procedures performed in this study were in accordance with the ethical standards of the institutional research committee and with the 1964 Helsinki Declaration and its later amendments. Informed consent was obtained from all individual participants included in the study. Local ethics committee approval was obtained (CEAS Umbria, n. 3592/19) and patients were enrolled in a longitudinal, prospective, randomized, controlled, clinical trial. We included patients with clinical diagnosis of viral conjunctivitis who presented to the Eye Casualty of the Hospital of Santa Maria della Misericordia in Perugia (Italy). All of them were fully evaluated at the slit lamp with fluorescein and the diagnosis was made based on the following criteria: presence of unilateral or bilateral bulbar redness, watery conjunctival discharge, follicles in the inferior conjunctival fornix and pre-auricular lymphadenopathy.

The patients were randomly assigned, according to a computer-generated sequence, in to two groups: group A (study group) and group B (control group). The former received topical tobramycin 0.3%/dexamethasone 0.1% eye drops (Combitimor, Sooft Italia S.p.a., Italy), plus ozonized oil eye drops (Ozodrop, FB Vision S.r.l., Italy) both four times daily while the latter topical tobramycin 0.3%/dexamethasone (Combitimor, Sooft Italia S.p.a., Italy) eye drops four times per day. The treatment was for seven days in both groups.

Conjunctival injection and conjunctival chemosis, graded on a 4-point clinical scale (trace, mild, moderate and severe), and the presence or absence of superficial punctate keratitis (SPK) and subepithelial corneal infiltrates were evaluated at the time of the diagnosis and at 7-day follow-up. For each patient a multiplex PCR real time analysis was carried out through two sterile conjunctival fornix swabs, one for each eye to avoid contaminations, for adenovirus on the day of the diagnosis and at 7-day follow-up. Conjunctival swabs of both eyes were taken at each visit using a sterile flocked swab kit (Copan Italia S.p.a., Brescia, Italy), rubbing the swab into the inferior conjunctival fornix for at least five seconds without touching the eyelashes or the eyelid skin to avoid any kind of contamination. No eye drops were applied prior the swab in order to preserve the vitality of the material taken. The samples were insert in a sterile container and quickly transferred to the microbiology laboratory for real time multiplex PCR*.* Negativization of the infection was defined when, in a patient with the first sample positive for adenovirus DNA by real time multiplex PCR, a second sample, obtained after 7 days of treatment, resulted negative for the same pathogen.

A chi-square test was used to compare treatment groups with respect to binary endpoints, the Mann–Whitney test was used for continuous variables and *P* values < 0.05 were considered statistically significant.

## Results

Eighty patients were enrolled in this study, 41 females and 39 males, mean age 40.5 years (range 19–72), and 40 patients were assigned in group A and 40 in group B. No differences were observed between the two groups at baseline and no patients were lost at follow-up, the treatment was well tolerated in both groups and none of the patients reported any treatment related adverse events.

At the baseline there was hyperemia and chemosis in all patients of both groups. The average value of hyperemia was 3.16 in Group A and 3.05 in Group B. Similarly, the average value of chemosis was 2.67 in Group A and 2.46 in Group B (Table 1).

**Table 1:** Pre-operative patients’ assessments

	Study group N. of Patients: 40 N. of Eyes: 80	Control group N. of Patients: 40 N. of Eyes: 80	All patients N. of Patients: 80 N. of Eyes: 160
Age (mean ± SD)	39.9 ± 14.6	41.1 ± 13.4	40.5 ± 14
Sex			
Male	18	21	39
Female	22	19	41

After 7 days of therapy, patients of both group showed an improvement in the clinical picture with reduction of hyperemia and chemosis of the conjunctiva, in particular after seven days of therapy, hyperemia was present in 55 eyes of Group A (average value 0.84) and in 66 eyes of Group B (average value 1.3) and the difference between the two groups was statistically significant. Similarly, chemosis was present at follow-up in 29 eyes in Group A (average value 0.36) and in 53 eyes in Group B (average value 0.82), and the difference between the two groups is statistically significant.

In group A, epitheliopathy was detected in 25 eyes at the baseline while after seven days of therapy was seen in 8 eyes; in group B it was found in 20 eyes at the baseline and on the seventh day in 10 eyes with no statistically differences between groups. After seven days of therapy, respectively 82.3% of group A and 59.4% of group B had a titer negativization while 6 patients of group A and 13 patients of group B were positive for PCR, with statistically significant differences between the groups (Table 2).

**Table 2:** Hypermia, chemosis, superficial punctate keratitis, subepithelial corneal infiltrates values and percentage of patients with positive PCR test of both groups at the two time-points of the study

	Study group		Control group	
	Baseline	7 day	Baseline	7 day
Eyes with hypermia	80	55	80	66
Hypermia (mean values)	3.05	0.84*	3.16	1.3*
N. of eyes with chemosis	80	29	80	53
Chemosis (mean values)	2.67	0.36**	2,46	0.82**
Eyes with SPK	25	8	20	10
Eyes with SEIs	2	6	1	6
Patients with positive PCR test (%)	34	6***	32	13***

*SPK* superficial punctate keratitis, *SEIs* subepithelial corneal infiltrates

^*^P < 0.05

^**^P < 0.05

^***^P < 0.05

## Discussion

The present study intended to evaluate the activity of ozonized oil eye drops plus tobramycin 0.3%/dexamethasone 0.1% eye drops compared with tobramycin 0.3%/dexamethasone 0.1% eye drops for clinical resolution of acute viral conjunctivitis. The results we observed showed that the combination of the two eye drops is safe and well tolerated and it has a better efficacy than tobramycin 0.3%/dexamethasone 0.1% eye drops for clinical resolution and adenoviral eradication.

Virus are responsible for over 80% of all infectious conjunctivitis and 65–90% are due to Adenoviruses (HAdV type 8,19,37,54) [16] which represents from 20 to 75% of all causes of infectious [17–20].

Adenoviral conjunctivitis is associated with significant patient discomfort, lost productivity, and is a highly infectious community epidemics which can lead to epidemic keratoconjunctivitis (EKC), usually caused by serotypes 8, 37, 64, considered public health concern [21–24]. The high contagiosity is due to the relative adenovirus resistance to disinfection and to his ability to survive in a desiccated state for several weeks at room temperature [2, 26]

Even though adenoviral conjunctivitis in almost all cases evolves without leaving effects within a few weeks, the conjunctival infection can affect the cornea 3–4 days later after conjunctivitis. The viral replication into the corneal epithelium manifests as a fine and diffuse epithelial keratitis resulting in focal areas of opacity which lasts for 2–3 weeks. About 2 weeks after the initial clinical manifestation multiple subepithelial corneal infiltrates can be seen in the anterior stroma, which appear as focal nubecules. Infection can involve the anterior part of the stroma with involvement of the keratocytes present here resulting in a focal immune reaction due to accumulation of lymphocytes, macrophages, monocytes and fibroblasts activated in response to corneal infection that clinically appears as nummular opacities in the anterior stroma with non-colorable cornea. These opacities may persist for weeks, months up to years are responsible for visual disturbances such as decreased vision, glare, photophobia and irregular astigmatism [22, 23, 25, 26]

The current management of EKC, in the absence of specific treatment, largely revolves around accurate diagnosis and implementation of disinfection protocol to prevent its spread: several therapeutic approaches have been proposed, to date there is no approved treatment for EKC. The American Academy of Ophthalmology guidelines advises on the use of artificial tears, topical antihistamines and steroids, oral analgesics, or cold compresses to mitigate symptoms [27].

Pelletier et al. in 2009 observed in a small number of adenoviral conjunctivitis patients that the use of povidone-iodine 0.4% and dexamethasone 0.1% ophthalmic suspension may be useful [28]. Recently, Kovalyuk et al. described that in patients affected with adenoviral keratoconjunctivitis the fastest improvement in red eyes, discharge, superficial punctate keratitis and pseudomembranes was in the group treated with povidone-iodine 1.0% and dexamethasone 0.1% eyedrops, compared to those treated with dexamethasone 0.1% or hypromellose 0.3% [29]. Moreover, some authors used Povidone-Iodine 0.6% and Dexamethasone 0.1% ophthalmic suspension in patients with acute adenoviral conjunctivitis reporting that its use was safe and well tolerated, significantly improving adenoviral eradication and clinical resolution [30]. The safety of this treatment in patients with clinically suspected acute viral conjunctivitis was recently confirmed by other authors who, however, failed to demonstrate its therapeutic superiority [31].

The use of the steroid in viral inflammations of the ocular surface does not agree with all the authors in fear of possible side effects such as cataract and glaucoma. Holland et al. underlines that the perceived risks associated with short-term corticosteroid use in the treatment of ocular surface infections (increased IOP, prolonged viral shedding, and HSV reactivation) is not supported by high-quality evidence in the literature [32]. However, it should not be forgotten that without the addition of a anti-viral agent can increase the duration of viral shedding and prolong the infectivity of the patients [12].

Oxidising agents that are commonly used to disinfect the skin and the solid surfaces, and ozone is known to be a powerful antibacterial, antiviral and antifungal agent and its antiseptic and anti-inflammatory properties are well known [33]. The ozone has been shown to be effective in accelerating acute cutaneous wound repair in a pig with the increased expression of PDGF, TGF-β and VEGF [34]. Murray et al. have highlighted the properties of the ozone proving its capability to reduce the viral infectivity by lipid peroxidation and subsequent lipid envelope and protein shell damage [35]. These data suggested that a wide range of pathogens can be inactivated in an environment of known ozone exposure. The use of ozone in some eye anterior segment pathologies could be providential due to its anti-inflammatory and bactericidal activity, in addition to promoting tissue repair properties. The safety of ozonated oil in liposomes has been assessed in vitro and in vivo. Cutarelli et al. demonstrated in vitro that this product showed no toxic effect on the keratinocyte, while preclinical evaluation have been shown that ozone is safe on the ocular surface [13, 15, 36, 37].

Authors have proven that the antibacterial activity of an ozonated oil is more effective antiseptic than chlorhexidine digluconate and povidone-iodine against *S. aureus* and the periodontal pathogen *P. gingivalis*.[38]. Spadea et al. have shown that ozone-based eye drops represent a valid and suitable alternative therapy for the management of external ocular pathologies in both animals and humans [13].

To the best of our knowledge our study is the first that demonstrate the efficacy and safety of ozonated oil eye drops in acute viral conjunctivitis: these drops along with the standard therapeutic scheme proved better than the standard treatment alone in controlling the ocular surface inflammation, obtaining a high percentage of negativizations of the previously positive conjunvtival swabs.

These data, in our opinion, are very interesting because antiseptic and antiviral activity of an ozonized oil in the treatment of viral conjunctivitis could be an original and important choice in the treatment of this important infection. In addition, it suggests new therapeutic prospects for ocular surface infections with the consequent possibility of reducing the use of topical antibiotic therapy, and therefore of the risk of antibiotic resistance.

Being a pilot study, our study has some limitations, first among all the relatively small number of patients. A second limitation is that patients with clinical diagnosis of viral conjunctivitis not confirmed by RT-PCR were included. Furthermore, it is well known that some virus serotypes induce more aggressive diseases than others: the failure to recognise the serotype might have influenced the results of this study. Lastly, the 7 days of treatment might have prevented us from understanding any long-term differences in the two groups of patients studied.

Further studies with larger numbers of patients and more controls are needed to confirm our observations by including a third control group that has been administered to tear substitute or eye drops vehicle.

Based on the results, we guess that our study is an additional incentive to consider the use of antiseptic eye drops in the treatment of infectious diseases of the ocular surface.
